# Beyond Lockdown: The Potential Side Effects of the SARS-CoV-2 Pandemic on Public Health

**DOI:** 10.3390/nu13051600

**Published:** 2021-05-11

**Authors:** Sara Paltrinieri, Barbara Bressi, Stefania Costi, Elisa Mazzini, Silvio Cavuto, Marta Ottone, Ludovica De Panfilis, Stefania Fugazzaro, Ermanno Rondini, Paolo Giorgi Rossi

**Affiliations:** 1 Physical Medicine and Rehabilitation Unit, Azienda Unità Sanitaria Locale-IRCCS di Reggio Emilia, 42123 Reggio Emilia, Italy; sara.paltrinieri@ausl.re.it (S.P.); barbara.bressi@ausl.re.it (B.B.); stefania.fugazzaro@ausl.re.it (S.F.); 2 Department of Biomedical, Metabolic and Neural Sciences, Università di Modena e Reggio Emilia, 41124 Modena, Italy; 3 Scientific Directorate, Azienda Unità Sanitaria Locale-IRCCS di Reggio Emilia, 42123 Reggio Emilia, Italy; 4 Department of Medicine, Surgery, Dentistry and Morphological Sciences, Università di Modena e Reggio Emilia, 41124 Modena, Italy; 5 Medical Directorate Hospital Network, Azienda Unità Sanitaria Locale-IRCCS di Reggio Emilia, 42123 Reggio Emilia, Italy; elisa.mazzini@ausl.re.it; 6 Clinical Trials and Statistics Unit, S.C. Infrastructure, Research and Statistics, Azienda Unità Sanitaria Locale-IRCCS di Reggio Emilia, 42123 Reggio Emilia, Italy; silvio.cavuto@ausl.re.it; 7 Epidemiology Unit, Azienda Unità Sanitaria Locale-IRCCS di Reggio Emilia, 42122 Reggio Emilia, Italy; marta.ottone@ausl.re.it (M.O.); paolo.giorgirossi@ausl.re.it (P.G.R.); 8 Bioethics Unit, Azienda Unità Sanitaria Locale-IRCCS di Reggio Emilia, 42123 Reggio Emilia, Italy; ludovica.depanfilis@ausl.re.it; 9 Lega Italiana contro i tumori-LILT Reggio Emilia, 42123 Reggio Emilia, Italy; ermanno.rondini@gmail.com; 10 Regional Center for Education in Health Promotion-Luoghi di Prevenzione, 42122 Reggio Emilia, Italy

**Keywords:** lifestyle, exercise, diet, alcohol drinking, cigarette smoking, COVID-19, quarantine, pandemics

## Abstract

Lockdowns to contain the spread of the SARS-CoV-2 have disrupted routines and behaviors, which could lead to a worsening of lifestyle and an increase in the burden of non-communicable diseases. This study aimed to describe the changes in physical activity, diet, alcohol drinking, and cigarette smoking during lockdown. A self-administered online survey addressing adults living in a province in northern Italy was advertised through websites and social media. Citizens could access the survey in anonymity from 4 May until 15 June 2020. A total of 1826 adults completed the survey, with a worsening of physical activity (35.1%), diet (17.6%), alcohol drinking (12.5%), and cigarette smoking (7.7%) reported. In contrast, 33.5% reported an improvement in diet, 12.6% in alcohol drinking, 5.3% in physical activity and 4.1% in cigarette smoking. Female sex, young adult age, suspension of work activity, and symptoms of psychological distress were the factors associated with a greater likelihood of change, which was frequently for the worse. Lockdown had an impact on lifestyle, with some net beneficial effects on diet and mostly negative effects on physical activity. Public health measures should be implemented to avoid long-term negative effects of the lockdown, supporting individuals more prone to change for the worse.

## 1. Introduction

The SARS-CoV-2 pandemic has had a tremendous direct impact on health, with over 2.5 million deaths registered worldwide by the end of February 2021 [1].

Further, indirect effects of the pandemic on public health are expected. A change in the routine care of non-communicable diseases (NCD) has become necessary in many countries to contain the spread of the SARS-CoV-2 virus, with a likely negative impact on their diagnosis, management, and progression [2,3,4]. Moreover, physical distancing and lockdown measures have disrupted individuals’ daily routines and behaviors [5], and it is widely believed that those changes have induced individuals to become more sedentary, adopt less healthy diets, and increase their alcohol and smoking consumption [6,7,8]. Because unhealthy behaviors are the main risk factor for chronic diseases and the major cause of disability and life years lost [9,10], physical distancing and mobility restrictions may reinforce these behaviors, thereby raising serious public health concern, particularly if such changes persist [11].

Understanding and quantifying the effect of lockdown on behaviors may help to identify strategies of outbreak containment and infection control that are less impactful or to implement actions that may lessen the negative impact of lockdown on health [2].

This is chiefly important for countries with a high life expectancy and a great burden of chronic diseases [12], and for those countries largely affected by the coronavirus outbreak, such as Italy, the first European nation where the pandemic peaked and that implemented lockdown [1]. In Italy, lockdown started on 11 March 2020 and lasted over two months. Restrictions were particularly harsh: all social, recreational, and production activities, except essential services for health and basic needs, were suspended. Educational activities continued online only and leaving one’s home for a walk was allowed only in the neighbourhood (within a radius of 200 m) [13]. The imperative to stay at home lasted until May 4, but most restrictions, such as travel restrictions or banned entry to gyms, among others, lasted up to the end of May.

Thus, we launched a cross-sectional study to investigate the lifestyle changes occurring during the lockdown in Italian adults living in Reggio Emilia, a province in Emilia-Romagna (northern Italy). Of the 20 Italian regions, Emilia-Romagna ranked in the top three for cumulative incidence of coronavirus infections during the spring 2020 wave of the pandemic [14]. The province of Reggio Emilia has a population of approximately 533,000 people, 66% of whom are between the ages of 18 and 70.

The aim of this study was to estimate changes in physical activity, diet, alcohol drinking, and cigarette smoking during lockdown. We also aimed to identify factors associated with changes in lifestyle.

## 2. Materials and Methods

### 2.1. Study Design, Participants, and Setting

This cross-sectional study was endorsed by the Azienda USL-IRCCS of Reggio Emilia (Italy). It employed the self-administration of an online survey addressing adults (aged ≥ 18) living in the province of Reggio Emilia, without restrictions.

### 2.2. Ethics

The study was conducted in accordance with European Regulation n.2016/679. According to Italian law, because the data collected were anonymous at the source, the Local Ethics Committee’s approval was not required. However, the survey was revised by an expert in ethics (LDP) prior to its dissemination. Participants were informed that personal data would not be collected and that they would be anonymized and used for research purposes exclusively. Eligible individuals could then voluntarily consent to participate in the survey, without prejudice to their right to stop at any stage before submission; responses were saved only by clicking the “submit” button.

The study was prospectively registered in ClinicalTrial.gov NCT04423978.

### 2.3. Procedures

A survey investigating the lifestyle components and any changes that occurred during lockdown was developed by a group of healthcare professionals made up of epidemiologists, physicians, and rehabilitation professionals. The survey was approved by the local branch of the Italian Cancer League-LILT (https://www.lilt.it/, accessed on 16, March 2021), a nonprofit association whose mission is cancer prevention that operates under the supervision of the Ministry of Health, and by the Regional Center for Multimedia Education for Health Promotion (https://www.luoghidiprevenzione.it/Home/, access on 16 March 2021) (Supplementary Material 1—S1). The survey included 49 questions exploring the following areas: (a) sociodemographic data (14 items); (b) work-related data (5 items); (c) computer literacy (3 items); (d) general health status and lifestyle prior to lockdown (23 items); (e) use of local social support services (3 items); (f) symptoms of psychological distress (1 item). Questions investigating lifestyle were based on the indexes used by the Italian National Institute of Health for the Italian behavioural risk factors surveillance system (PASSI) [15], which is based on the United States CDC’s Behavioral Risk Factor Surveillance System [16]. The questions were formulated in such a way as to bring out the changes that occurred during lockdown.

Apart from two open-ended questions, the answers were multiple choice, with more than one choice possible in some cases. Due to the time constraints associated with the temporary lockdown, the questionnaire did not undergo formal validation. The survey took an average of 15 min to complete; answering all the questions was not mandatory.

On May 4, the survey was publicised on the websites and social media of the Azienda USL-IRCCS of Reggio Emilia, the major municipalities of the province, the network of the municipal pharmacies, and the local patient associations that joined the initiative. Citizens could access the survey in complete anonymity until 15 June 2020.

### 2.4. Outcomes

The outcome of this study is the self-reported change in lifestyle components during the COVID-19 lockdown: physical activity, diet, alcohol drinking, and cigarette smoking.

For physical activity, we asked participants whether they were sedentary, partially active, or active both before and during lockdown. Based on the answers, changes in physical activity were categorized as “improved”, “worsened”, and “unchanged” (questions 27 and 29 of the Supplementary Material 1).

For diet, we asked participants whether their diet had changed during lockdown and which changes had taken place (questions 33, 34, 35, and 36 of Supplementary Material 1). Diet was then categorized as “improved”, “worsened”, “unchanged”, and “mixed behaviors”. This latter category included changes in diet that were both in the direction of a more and of a less healthy diet (e.g., eating more fruit and vegetables but also drinking more carbonated drinks). In the multivariate analysis, we grouped the mixed and unchanged behaviors in one category, used as reference.

Changes in alcohol drinking and cigarette smoking were categorized as “decreased”, “increased”, and “unchanged” (questions 39 and 41 of Supplementary Material 1).

### 2.5. Potential Determinants and Covariates

We also collected data on sociodemographic factors (sex, age, education level, household composition), work-related factors (occupational status and changes due to the lockdown), health status and lifestyle prior to lockdown (presence of chronic diseases, body mass index, physical activity habits, alcohol drinking habits, cigarette smoking habits) and symptoms of psychological distress (feeling upset, tension, worry, fear, loneliness, and/or uncertainty). The data were used to verify any associations between these potential determinants and the outcomes of interest.

### 2.6. Analyses

To verify whether and how the responding sample differed from the population living in the same province, we compared its distribution by age and sex with that of the resident population (Table 1).

We also compared the age-adjusted proportions observed in our sample for education level, household composition, economic difficulties, BMI, physical activity, alcohol drinking, and cigarette smoking with those of the resident population based on the data from the Italian Health Interview Survey (18–69 years) [15], that used a representative sampling procedure.

We report the proportion of all the lifestyle changes based on sociodemographic information, work-related factors, health status, and lifestyle prior to lockdown and symptoms of psychological distress as potential exposures. Then we built multinomial models (logistic regression models), that use a general logit as link function, to assess the association between the potential determinants and positive or negative changes. Odds ratios produced by the models were adjusted for sex, age, and education level, while other variables were included one by one. Since the causal relationships between other putative determinants are not known and because, in many cases, they may act along the same causal pathway, we decided not to include them simultaneously in multivariate models. In fact, if two factors are consecutive links in the same causal chain, putting them together in the same model would hide the association between each upstream determinant in the causal chain and the outcome. Multivariate analyses were performed using SAS System, version 9.4 for Windows OS.

No predefined significance threshold was defined; given the exploratory nature of the study, we did not perform any formal statistical test of hypothesis. Investigated associations were evaluated for their precision, and the probability of being due to chance according to 95% confidence intervals (CI) and *p*-values are to be interpreted as a continuous variable.

## 3. Results

### 3.1. Participants’ Characteristics

A total of 1826 individuals completed the online survey, which constituted 0.36% of the adult residents in the province of Reggio Emilia.

Table 1 describes the distribution of exposures stratified by sex. Females, young and middle-aged adults, individuals with a high education level, those living with at least one person, and those reporting no economic difficulties were overrepresented. Slightly more than 80% of the participants reported no chronic diseases. The distribution of BMI in the sample was similar to that of the general population of the same age. Finally, respondents showed healthier behaviors in terms of physical activity, alcohol drinking, and cigarette smoking.

### 3.2. Lifestyle Changes during Lockdown

Table 2, Table 3, Table 4 and Table 5 report the descriptive statistics and the odds ratios with their confidence intervals concerning the changes in physical activity, diet, alcohol drinking, and cigarette smoking.

During the lockdown, 40.4% of participants changed their physical activity habits: 35.1% had a decrease, while 5.3% an increase. More than half of the participants changed their diet; in 17.6% of cases, these changes were for the worse (e.g., eating more snacks, sweets, carbonated drinks), while in 33.5%, they were improvements (e.g., paying more attention to eating healthier). Changes in alcohol drinking occurred in both directions equally, since 12.5% of individuals increased their alcohol consumption and 12.6% decreased it. Cigarette smoking changed for 11.8% of participants, of whom 7.7% reported an increase and 4.1% a decrease.

Supplementary Material 2 (S2) reports the co-occurrence of more than one negative change in lifestyle. In the whole sample, 7.9% of participants reported a worsening in both physical activity and dietary habits; this proportion was only slightly higher than the expected proportion in the hypothesis of independence, as these two worsening behaviors were not associated at all, i.e., 6.2%. Furthermore, about 2% declared a negative change also in alcohol drinking habits.

### 3.3. Influence of Potential Determinants on Lifestyle Changes

Table 2, Table 3, Table 4 and Table 5 also report sociodemographic and work-related factors, health status, lifestyle prior to lockdown, and symptoms of psychological distress associated with changes in lifestyle, and the Supplementary Materials 3 (S3) summarizes these results.

Being female was more likely associated with a change in physical activity habits in both directions (OR 1.96, CI 1.08–3.53 for improvement, OR 1.22, CI 0.96–1.56 for worsening) and with a worsening of diet (OR 1.90, CI 1.33–2.71). Additionally, females less frequently decreased their alcohol consumption (OR 0.47, CI 0.34–0.65). Compared to adults aged 18 to 44, participants aged ≥ 65 years were less likely to change their lifestyle (see Table 2, Table 3 and Table 4), except for the few who increased cigarette smoking (*n* = 23, accounting for 11.9% of participants over age 65). Compared to participants with a low education level, those with a higher education level were more likely to improve their diet (OR 1.22, CI 0.72–2.06 for medium education, OR 1.57, CI 0.93–2.66 for high education), but a clear trend in one direction was not identifiable for each outcome, as it was for cigarette smoking (see Table 5), furthermore observed differences are compatible with random fluctuations. People living with at least one cohabitant were less likely to decrease alcohol consumption and cigarette smoking (OR 0.55, CI 0.36–0.85 and OR 0.48, CI 0.25–0.93, respectively). Overall, being able to continue working positively affected lifestyle, since both working in the usual modalities or remote working lessened the likelihood of worsening physical activity (OR 0.50, CI 0.31–0.79 for usual modalities, OR 0.60, CI 0.38–0.97 for remote working) and diet (OR 0.52, CI 0.30–0.89 for usual modalities, OR 0.51, CI 0.30–0.89 for remote working). Overweight individuals were more likely to improve their physical activity and alcohol drinking habits and to worsen their diet compared to normal weight individuals (see Table 2; Table 4). Smokers and sedentary individuals prior to lockdown were more likely to improve their physical activity habits compared both to non-smokers and partially active individuals (see Table 2). Finally, participants who did not report any symptoms of psychological distress were less likely to worsen their lifestyle, but the absence of some of those symptoms did not always protect against a consistent worsening in lifestyle. For instance, not perceiving fear seemed to protect against a worsening in diet (OR 0.65, CI 0.47–0.90) and alcohol consumption (OR 0.58, CI 0.40–0.83), but its effect on physical activity or smoking habits is uncertain (see Table 2; Table 5).

## 4. Discussion

This cross-sectional survey showed that, in a self-selected sample of Italian adults, the lockdown in the spring of 2020 led to a change in lifestyle, particularly in physical activity and diet. However, while changes in diet also saw the adoption of healthier behaviors, physical activity changed mostly for the worse. Alcohol consumption changed equally in both directions, and cigarette smoking showed a predominantly increasing trend.

Negative changes did not show any strong associations with each other, and only 7.9% of the respondents showed a worsening in both diet and physical activity.

Surprisingly, while psychological distress had a substantially negative effect on all changes, none of the other potential determinants that were investigated had a clear effect on physical activity, diet, drinking, or smoking.

Clearly, physical activity was the lifestyle component that showed the strongest net negative impact of the lockdown. This is probably because in Italy, in contrast to other countries affected by the pandemic, the restrictions imposed during lockdown extended to outdoor physical activities.

This study adds knowledge concerning the changes in lifestyle occurring during lockdown, and even though similar surveys have been conducted in Italy also at national level, our own results suggest that there are vulnerable individuals who may have been more prone to changes for the worse.

To date, several cross-sectional studies have postulated the negative impact of lockdown on the lifestyle of adult individuals living in countries highly affected by the SARS-CoV-2 virus. Beyond the results of the individual studies, none of which can be considered exhaustive, similar overall effects on lifestyle were observed: the lockdown triggered a trend towards increased sedentariness and weight gain [17,18,19,20,21], which was often associated with unhealthy eating patterns [17,18,22] that were more characteristic of females [17], although greater care in choosing healthy foods was also observed [20,21,22]. Tobacco smoking may or may not have increased during lockdown [17,21,22,23], while the overall trend for alcohol consumption seemed to increase [17,22]. However, all the studies conducted on this theme show that, for each of the lifestyle components, there were individuals whose habits worsened but also those with resilient attitudes, i.e., capable of taking advantage of social isolation to improve their lifestyle. Thus, the lockdown triggered changes in lifestyle, but while some individuals managed to steer these changes for the better, many others did not. This was particularly true for physical activity, which the literature has reported as having decreased. Thus, individuals at major risk of adopting unhealthy behaviors should be identified, and public health interventions should target these groups to prevent a lapse or to support the return to healthy habits once the lockdown is over. These interventions are justified, because unhealthy behaviors are the main risk factor for developing non-communicable diseases [9,10].

Who are the individuals at the greatest risk of a worsening in lifestyle during lockdown?

Based on our results, female sex, young adult age, suspension of work activity, and symptoms of psychological distress were the factors associated with a greater likelihood of change, which was frequently for the worse.

In fact, women seemed to be more affected than men: their diet and, although not consistently, their physical activity habits are more likely to worsen. These results are consistently in line with the evidence in the previous cross-sectional surveys conducted during the spring 2020 wave of the pandemic. Danish females were prone to more snacking and to gain in weight [24], and about half of those interviewed in Saudi Arabia by Al-Musharaf have reported moderate or high level of emotional eating [25]. In contrast, Italian men shown a healthier diet [17].

Age over 65 seemed to protect against a worsening in lifestyle, at least for non-smoker Italians. This finding is consistent with those of Ferrante and collaborators [17], who demonstrated that older individuals did not seem to increase alcohol intake. Nevertheless, another Italian survey reported that young adults seemed to adhere better to a Mediterranean diet when compared to older individuals [21].

Work suspension may have exposed individuals to a worsening in both physical activity and dietary habits, as also shown by Di Renzo and collaborators [21], who reported that employees who suspended work perceived an increase in weight and a change of appetite. This worsening may be justified by the abrupt disruption of daily routines but also by the economic effects of the pandemic, which arouse uncertainty about the future as well as sleep disturbances [26]. In fact, our results showed that symptoms of psychological distress perceived during lockdown were consistently associated with worsening of lifestyle. For adults living in the USA, weight gain was associated with higher levels of psychological distress, which persisted for months after the spring lockdown [27].

Moreover, although our data showed that overweight individuals were more likely to improve their physical activity and alcohol drinking habits, they also showed that the diet of these same individuals worsened compared to that of normal weight individuals.

This result is consistently in line with that of a global survey carried out during the spring 2020 wave by Flanagan and collaborators, who showed that obese individuals reported a gain in weight [20]. Thus, as strongly claimed in the literature [20,28], overweight individuals should be monitored to prevent a further deterioration of their health conditions during a lockdown.

### Limitations

The main limitation of this study is that our sample was not representative of the resident population. The sampling modality (voluntary participation) and the dissemination and questionnaire administration strategies (through institutional websites) selected a sample that was younger, with higher proportions of females and individuals with a high education level and healthier habits compared to that of the resident population, even when taking into account differences in age and sex [15]. Additionally, although the rate of missing data was low for most of the items investigated, it was almost 10% in a few cases, thus reducing the precision of the estimates made. More importantly, the data collected covered only self-perceived phenomena, and collection relied on a questionnaire that had not been previously validated. It is therefore likely that the self-administered modality of the questionnaire may have led to the so-called social desirability bias, i.e., the over-reporting of perceived virtuous behaviors to achieve social approval [29]. Thus, the results we obtained must be interpreted in light of all these potential sources of bias. However, it must be considered that during the spring 2020 wave of the pandemic, this was the only feasible sampling method of the population. Further, similar cross-sectional surveys were widely implemented by health authorities and national surveillance systems all over the world, and their results often converge with ours. Finally, we reported more than 50 comparisons between different groups for their changes in lifestyles, for all of them we measured the association, and we computed the probability that the association would be due to chance, i.e., the *p*-value; given the high number of comparison, it is therefore very likely that some of the observed associations showed a small *p*-value only by chance, even in the absence of any true association.

## 5. Conclusions

The collateral damage of the lockdown on individuals with NCD has been extensively substantiated in the literature, highlighting the expected effects of delayed diagnoses and treatments as well as the detrimental effects of physical distancing on caring for family members, and the risk of individuals’ developing mood disturbances [30]. Additionally, the lockdown also triggered changes in lifestyle, and although the long-lasting effects of these changes have not been verified, the negative impact of the worsening of lifestyle habits—physical activity in particular—is very likely. Therefore, a wave of long-term negative effects may occur if the negative changes of the lockdown on lifestyle are not counteracted. With this specific aim, public health measures should be implemented during and beyond a lockdown which target chiefly the vulnerable individuals, more prone to change for the worse, that this study has contributed to identifying.

## Figures and Tables

**Table 1:** Descriptive analysis of participants by sex: sociodemographic factors, work-related factors, health status, and lifestyle prior to lockdown.

						**Sample of Participants**		**Resident Population**	
		**Total**	**Male**	**Female**	**Missing**	**Male**	**Female**	**Male**	**Female**
		*n* (%)	*n* (%)	*n* (%)	*n* (%)	% observed		% expected	
		1826 (100)	423 (23.2)	1397 (76.5)	6 (0.3)	21.2 *	72.5 *	49.2 **	50.8 **
**Sociodemographic factors**									
Age	Young adults (18–44)	818 (44.8)	182 (22.2)	636 (77.8)	0 (0.0)	··	··	31 **	
	Middle-aged (45–64)	802 (43.9)	179 (22.3)	622 (77.6)	1 (0.1)	··	··	30 **	
	Aged (≥65)	194 (10.6)	62 (32.0)	132 (68.0)	0 (0.0)	··	··	21.7 **	
	Missing	12 (0.7)	0 (0.0)	7 (58.3)	5 (41.7)	··	··	··	
Education level	Low	94 (5.1)	32 (34.0)	62 (66.0)	0 (0.0)	6.2 *	3.8 *	35.3 *	28.0 *
	Medium	805 (44.1)	190 (23.6)	614 (76.3)	1 (0.1)	45.6 *	45.2 *	47.4 *	48.4 *
	High	889 (48.7)	194 (21.8)	695 (78.2)	0 (0.0)	48.2 *	51.1 *	17.3 *	23.5 *
	Missing	38 (2.1)	7 (18.4)	26 (68.4)	5 (13.2)	··	··	··	··
Household composition	Alone	208 (11.4)	51 (24.5)	156 (75.0)	1 (0.5)	··	··	36.0 **	
	At least one cohabitant	1618 (88.6)	372 (23.0)	1241 (76.7)	5 (0.3)	··	··	64.0 **	
**Work-related factors**									
Changes in work modality	Work suspended	103 (5.6)	14 (13.6)	88 (85.4)	1 (1.0)	··	··	··	··
	More remote working	544 (29.8)	128 (23.5)	416 (76.5)	0 (0.0)	··	··	··	··
	Unchanged	685 (37.5)	168 (24.5)	516 (75.3)	1 (0.1)	··	··	··	··
	Not applicable	313 (17.1)	81 (25.9)	231 (73.8)	1 (0.3)	··	··	··	··
	Missing	181 (9.9)	32 (17.7)	146 (80.7)	3 (1.7)	··	··	··	··
Economic difficulties	No	1320 (72.3)	317 (24.0)	1001 (75.8)	2 (0.2)	76.4 *	74.1 *	67.0 *	60.7 *
	Some	399 (21.9)	83 (20.8)	316 (79.2)	0 (0.0)	21.6 *	23.7 *	26.7 *	31.7 *
	Many	38 (2.1)	9 (23.7)	29 (76.3)	0 (0.0)	2.1 *	2.2 *	6.3 *	7.6 *
	Missing	69 (3.8)	14 (20.3)	51 (73.9)	4 (5.8)	··	··	··	··
**Health status and lifestyle prior to lockdown**									
Presence of chronic diseases	No	1506 (82.5)	355 (23.6)	1145 (76.0)	6 (0.4)	··	··	··	··
	Yes	320 (17.5)	68 (21.3)	252 (78.8)	0 (0.0)	··	··	··	··
BMI ***	Normal weight	1052 (57.6)	204 (19.4)	845 (80.3)	3 (0.3)	52.8 *	67.9 *	48.8 *	67.7 *
	Overweight	600 (32.9)	192 (32.0)	408 (68.0)	0 (0.0)	47.2 *	32.1 *	51.2 *	32.3 *
	Missing	174 (9.5)	27 (15.5)	144 (82.8)	3 (1.7)	··	··	··	··
Physical activity habits	Sedentary	239 (13.1)	45 (18.8)	194 (81.2)	0 (0.0)	11.5 *	14.0 *	13.3 *	19.5 *
	Partially active	995 (54.5)	202 (20.3)	791 (79.5)	2 (0.2)	47.4 *	57.9 *	26.3 *	31.4 *
	Active	548 (30.0)	169 (30.8)	379 (69.2)	0 (0.0)	41.0 *	28.1 *	60.4 *	49.0 *
	Missing	44 (2.4)	7 (15.9)	33 (75.0)	4 (9.1)	··	··	··	··
Alcohol drinking habits	Not a drinker	824 (45.1)	141 (17.1)	683 (82.9)	0 (0.0)	32.4 *	48.4 *	20.0 *	40.9 *
	Moderate drinker	780 (42.7)	230 (29.5)	549 (70.4)	1 (0.1)	67.6 *	51.6 *	80.0 *	59.1 *
	High-risk drinker	176 (9.6)	48 (27.3)	127 (72.2)	1 (0.6)				
	Missing	46 (2.5)	4 (8.7)	38 (82.6)	4 (8.7)	··	··	··	··
Cigarette smoking habits	Smoker	389 (21.3)	95 (24.4)	294 (75.6)	0 (0.0)	24.0 *	21.9 *	30.4 *	22.9 *
	Non-smoker	1195 (65.4)	269 (22.5)	926 (77.5)	0 (0.0)	64.4 *	68.5 *	44.9 *	58.9 *
	Former smoker	189 (10.4)	52 (27.5)	135 (71.4)	2 (1.1)	11.6 *	9.6 *	24.7 *	18.1 *
	Missing	53 (2.9)	7 (13.2)	42 (79.2)	4 (7.5)	··	··	··	··

* Comparison between the age-adjusted proportions observed in our sample and those of the resident population. ** Proportions of participants expected, based on the website of the province of Reggio Emilia. *** Body mass index = BMI < 25 normal weight; BMI ≥ 25 overweight.

**Table 2:** Odds ratio for type of change in physical activity (improved, worsened, or unchanged) during COVID-19 lockdown by sociodemographic and work-related factors, health status, and lifestyle prior to lockdown, and psychological distress.

		**Total**	**Improved**	**Worsened**	**Unchanged**	**Missing**	**Improved**			**Worsened**			
		*n* (%)					ORs	95% CI		ORs	95% CI		
								lower limit	upper limit		lower limit	upper limit	*p* value
		1826 (100.0)	97 (5.3)	641 (35.1)	972 (53.2)	116 (6.4)							
**Sociodemographic factors**													
Sex	Male	423 (23.2)	15 (3.5)	140 (33.1)	253 (59.8)	15 (3.5)	1	..	..	1	..	..	0.035
	Female	1397 (76.5)	82 (5.9)	500 (35.8)	718 (51.4)	97 (6.9)	1.96	1.08	3.53	1.22	0.96	1.56	
	Missing	6 (0.3)	0 (0.0)	1 (16.7)	1 (16.7)	4 (66.7)	..	..	..	..	..	..	
Age *	Young adults	818 (44.8)	64 (7.8)	302 (36.9)	409 (50.0)	43 (5.3)	1	..	..	1	..	..	<0.001
	Middle-aged	802 (43.9)	27 (3.4)	287 (35.8)	447 (55.7)	41 (5.1)	0.40	0.24	0.64	0.88	0.71	1.10	
	Aged	194 (10.6)	5 (2.6)	48 (24.7)	113 (58.2)	28 (14.4)	0.28	0.10	0.79	0.64	0.44	0.94	
	Missing	12 (0.7)	1 (8.3)	4 (33.3)	3 (25.0)	4 (33.3)	..	..	..	..	..	..	
Education level	Low	94 (5.1)	1 (1.1)	26 (27.7)	60 (63.8)	7 (7.4)	1	..	..	1	..	..	0.312
	Medium	805 (44.1)	41 (5.1)	282 (35.0)	402 (49.9)	80 (9.9)	4.53	0.61	33.91	1.51	0.92	2.48	
	High	889 (48.7)	54 (6.1)	322 (36.2)	488 (54.9)	25 (2.8)	3.98	0.53	29.81	1.38	0.83	2.27	
	Missing	38 (2.1)	1 (2.6)	11 (28.9)	22 (57.9)	4 (10.5)	..	..	..	..	..	..	
Household composition	Alone	208 (11.4)	11 (5.3)	73 (35.1)	115 (55.3)	9 (4.3)	1	..	..	1	..	..	0.898
	At least one cohabitant	1618 (88.6)	86 (5.3)	568 (35.1)	857 (53.0)	107 (6.6)	0.85	0.44	1.67	0.98	0.71	1.35	
**Work-related factors**													
Changes in work modality	Work suspended	103 (5.6)	6 (5.8)	50 (48.5)	40 (38.8)	7 (6.8)	1	..	..	1	..	..	0.001
	More remote working	544 (29.8)	47 (8.6)	200 (36.8)	274 (50.4)	23 (4.2)	1.17	0.46	2.98	0.60	0.38	0.97	
	Unchanged	685 (37.5)	24 (3.5)	230 (33.6)	398 (58.1)	33 (4.8)	0.48	0.18	1.27	0.50	0.31	0.79	
	Not appl. **	313 (17.1)	15 (4.8)	84 (26.8)	180 (57.5)	34 (10.9)	0.96	0.33	2.80	0.44	0.26	0.76	
	Missing	181 (9.9)	5 (2.8)	77 (42.5)	80 (44.2)	19 (10.5)	..	..	..	..	..	..	
**Health status and lifestyle prior to lockdown**													
Body mass index	Overweight	600 (32.9)	37 (6.2)	210 (35.0)	310 (51.7)	43 (7.2)	1	..	..	1	..	..	0.054
	Normal weight	1052 (57.6)	55 (5.2)	376 (35.7)	577 (54.8)	44 (4.2)	0.58	0.37	0.92	0.87	0.69	1.10	
	Missing	174 (9.5)	5 (2.9)	55 (31.6)	85 (48.9)	29 (16.7)	..	..	..	..	..	..	
Physical activity habits ***	Sedentary	239 (13.1)	47 (19.7)	0 (0.0)	180 (75.3)	12 (5.0)	1	..	..	1	..	..	<0.001
	Partially active	995 (54.5)	50 (5.0)	355 (35.7)	543 (54.6)	47 (4.7)	0.33	0.21	0.52	-	-	-	
	Active	548 (30.0)	0 (0.0)	286 (52.2)	249 (45.4)	13 (2.4)	-	-	-	-	-	-	
	Missing	44 (2.4)	0 (0.0)	0 (0.0)	0 (0.0)	44 (100.0)	..	..	..	..	..	..	
Cigarette smoking habits	Smoker	389 (21.3)	34 (8.7)	138 (35.5)	183 (47.0)	34 (8.7)	1	..	..	1	..	..	0.014
	Non-smoker	1195 (65.4)	53 (4.4)	436 (36.5)	655 (54.8)	51 (4.3)	0.44	0.28	0.72	0.89	0.69	1.15	
	Former smoker	189 (10.4)	9 (4.8)	55 (29.1)	112 (59.3)	13 (6.9)	0.59	0.27	1.31	0.73	0.49	1.09	
	Missing	53 (2.9)	1 (1.9)	12 (22.6)	22 (41.5)	18 (34.0)	..	..	..	..	..	..	
**Symptoms of Psychological distress**													
Tension	Yes	359 (19.7)	19 (5.3)	162 (45.1)	153 (42.6)	25 (7.0)	1	..	..	1	..	..	<0.001
	No	1337 (73.2)	74 (5.5)	445 (33.3)	751 (56.2)	67 (5.0)	0.91	0.53	1.57	0.59	0.45	0.76	
	Missing	130 (7.1)	4 (3.1)	34 (26.2)	68 (52.3)	24 (18.5)	..	..	..	..	..	..	
Upset	Yes	372 (20.4)	20 (5.4)	156 (41.9)	178 (47.8)	18 (4.8)	1	..	..	1	..	..	0.030
	No	1284 (70.3)	71 (5.5)	435 (33.9)	725 (56.5)	53 (4.1)	0.94	0.55	1.60	0.71	0.55	0.92	
	Missing	170 (9.3)	6 (3.5)	50 (29.4)	69 (40.6)	45 (26.5)	..	..	..	..	..	..	
Worry	Yes	810 (44.4)	37 (4.6)	323 (39.9)	406 (50.1)	44 (5.4)	1	..	..	1	..	..	0.002
	No	914 (50.1)	52 (5.7)	291 (31.8)	527 (57.7)	44 (4.8)	1.13	0.71	1.78	0.70	0.57	0.86	
	Missing	102 (5.6)	8 (7.8)	27 (26.5)	39 (38.2)	28 (27.5)	..	..	..	..	..	..	
Fear	Yes (ref)	303 (16.6)	13 (4.3)	115 (38.0)	150 (49.5)	25 (8.3)	1	..	..	1	..	..	0.329
	No	1404 (76.9)	81 (5.8)	490 (34.9)	768 (54.7)	65 (4.6)	1.28	0.68	2.38	0.86	0.65	1.13	
	Missing	119 (6.5)	3 (2.5)	36 (30.3)	54 (45.4)	26 (21.8)	..	..	..	..	..	..	
Loneliness	Yes (ref)	249 (13.6)	13 (5.2)	110 (44.2)	116 (46.6)	10 (4.0)	1	..	..	1	..	..	0.028
	No	1439 (78.8)	79 (5.5)	493 (34.3)	798 (55.5)	69 (4.8)	0.88	0.47	1.65	0.67	0.50	0.90	
	Missing	138 (7.6)	5 (3.6)	38 (27.5)	58 (42.0)	37 (26.8)	..	..	..	..	..	..	
Uncertainty	Yes	996 (54.5)	55 (5.5)	405 (40.7)	482 (48.4)	54 (5.4)	1	..	..	1	..	..	<0.001
	No	778 (42.6)	39 (5.0)	222 (28.5)	470 (60.4)	47 (6.0)	0.78	0.50	1.21	0.57	0.46	0.70	
	Missing	52 (2.8)	3 (5.8)	14 (26.9)	20 (38.5)	15 (28.8)	..	..	..	..	..	..	

ORs are adjusted for age, sex and education level. Age is adjusted for sex and education level; sex is adjusted for age and education level; education level is adjusted for age and sex. * Young adults (18–44); middle-aged (45–64); aged (≥65). ** Not appl. = participants who were retired, students, or housewives before COVID-19 lockdown. *** Due to few or null events in one or more covariate patterns, it was impossible to estimate model parameters.

**Table 3:** Odds ratio for type of change in diet (improved, worsened, mixed behaviors, or unchanged) during COVID-19 lockdown by sociodemographic and work-related factors, health status, and lifestyle prior to lockdown, and psychological distress.

		**Total**	**Improved**	**Worsened**	Mixed Behaviors *	Unchanged *	**Missing**	**Improved**			**Worsened**			
		*n* (%)						ORs	95% CI		ORs	95% CI		*p* value
									lower limit	upper limit		lower limit	upper limit	
		1826 (100.0)	612 (33.5)	321 (17.6)	337 (18.5)	530 (29.0)	26 (1.4)							
**Sociodemographic factors**														
Sex	Male	423 (23.2)	156 (36.9)	48 (11.3)	60 (14.2)	150 (35.5)	9 (2.1)	1	..	..	1	..	..	<0.001
	Female	1397 (76.5)	456 (32.6)	272 (19.5)	277 (19.8)	379 (27.1)	13 (0.9)	0.90	0.71	1.15	1.90	1.33	2.71	
	Missing	6 (0.3)	0 (0.0)	1 (16.7)	0 (0.0)	1 (16.7)	4 (66.7)	..	..	..	..	..	..	
Age **	Young adult	818 (44.8)	289 (35.3)	165 (20.2)	168 (20.5)	187 (22.9)	9 (1.1)	1	..	..	1	..	..	<0.001
	Middle-aged	802 (43.9)	274 (34.2)	136 (17.0)	132 (16.5)	250 (31.2)	10 (1.2)	0.95	0.76	1.19	0.70	0.52	0.92	
	Aged	194 (10.6)	45 (23.2)	18 (9.3)	36 (18.6)	92 (47.4)	3 (1.5)	0.44	0.30	0.66	0.30	0.17	0.51	
	Missing	12 (0.7)	4 (33.3)	2 (16.7)	1 (8.3)	1 (8.3)	4 (33.3)	..	..	..	..	..	..	
Educational level	Low	94 (5.1)	23 (24.5)	17 (18.1)	19 (20.2)	34 (36.2)	1 (1.1)	1	..	..	1	..	..	<0.001
	Medium	805 (44.1)	242 (30.1)	163 (20.2)	162 (20.1)	227 (28.2)	11 (1.4)	1.22	0.72	2.06	0.96	0.53	1.73	
	High	889 (48.7)	338 (38.0)	135 (15.2)	148 (16.6)	259 (29.1)	9 (1.0)	1.57	0.93	2.66	0.67	0.37	1.24	
	Missing	38 (2.1)	9 (23.7)	6 (15.8)	8 (21.1)	10 (26.3)	5 (13.2)	..	..	..	..	..	..	
Household composition	Alone	208 (11.4)	64 (30.8)	43 (20.7)	30 (14.4)	68 (32.7)	3 (1.4)	1	..	..	1	..	..	0.213
	At least one cohabitant	1618 (88.6)	548 (33.9)	278 (17.2)	307 (19.0)	462 (28.6)	23 (1.4)	1.01	0.72	1.42	0.72	0.48	1.07	
**Work-related factors**														
Changes in work modality	Work suspended	103 (5.6)	32 (31.1)	33 (32.0)	23 (22.3)	14 (13.6)	1 (1.0)	1	..	..	1	..	..	0.002
	More remote working	544 (29.8)	219 (40.3)	88 (16.2)	106 (19.5)	122 (22.4)	9 (1.7)	1	0.60	1.69	0.51	0.30	0.89	
	Unchanged	685 (37.5)	200 (29.2)	132 (19.3)	125 (18.2)	225 (32.8)	3 (0.4)	0.63	0.38	1.06	0.52	0.30	0.89	
	Not applicable	313 (17.1)	91 (29.1)	42 (13.4)	42 (13.4)	133 (42.5)	5 (1.6)	0.86	0.48	1.54	0.50	0.27	0.94	
	Missing	181 (9.9)	70 (38.7)	26 (14.4)	41 (22.7)	36 (19.9)	8 (4.4)	..	..	..	..	..	..	
**Health status and lifestyle prior to lockdown**														
Body mass index	Overweight	600 (32.9)	208 (34.7)	120 (20.0)	119 (19.8)	149 (24.8)	4 (0.7)	1	..	..	1	..	..	0.010
	Normal weight	1052 (57.6)	361 (34.3)	168 (16.0)	173 (16.4)	335 (31.8)	15 (1.4)	0.82	0.64	1.04	0.64	0.48	0.86	
	Missing	174 (9.5)	43 (24.7)	33 (19.0)	45 (25.9)	46 (26.4)	7 (4.0)	..	..	..	..	..	..	
Physical activity habits	Sedentary	239 (13.1)	76 (31.8)	44 (18.4)	40 (16.7)	77 (32.2)	2 (0.8)	1	..	..	1	..	..	0.463
	Partially active	995 (54.5)	346 (34.8)	180 (18.1)	194 (19.5)	265 (26.6)	10 (1.0)	1.17	0.84	1.61	1.10	0.74	1.63	
	Active	548 (30.0)	176 (32.1)	91 (16.6)	96 (17.5)	178 (32.5)	7 (1.3)	0.96	0.67	1.36	0.89	0.58	1.37	
	Missing	44 (2.4)	14 (31.8)	6 (13.6)	7 (15.9)	10 (22.7)	7 (15.9)	..	..	..	..	..	..	
Cigarette smoking habits	Smoker	389 (21.3)	116 (29.8)	88 (22.6)	85 (21.9)	97 (24.9)	3 (0.8)	1	..	..				0.126
	Non- smoker	1195 (65.4)	427 (35.7)	194 (16.2)	207 (17.3)	357 (29.9)	10 (0.8)	1.16	0.88	1.53	0.74	0.54	1.01	
	Former smoker	189 (10.4)	57 (30.2)	32 (16.9)	38 (20.1)	60 (31.7)	2 (1.1)	1.02	0.67	1.54	0.78	0.48	1.29	
	Missing	53 (2.9)	12 (22.6)	7 (13.2)	7 (13.2)	16 (30.2)	11 (20.8)	..	..	..	..	..	..	
**Symptoms of Psychological distress**														
Tension	Yes	359 (19.7)	97 (27.0)	105 (29.2)	78 (21.7)	76 (21.2)	3 (0.8)	1	..	..	1	..	..	<0.001
	No	1337 (73.2)	479 (35.8)	197 (14.7)	238 (17.8)	408 (30.5)	15 (1.1)	1.16	0.87	1.55	0.50	0.37	0.69	
	Missing	130 (7.1)	36 (27.7)	19 (14.6)	21 (16.2)	46 (35.4)	8 (6.2)	..	..	..	..	..	..	
Upset	Yes	372 (20.4)	126 (33.9)	87 (23.4)	81 (21.8)	75 (20.2)	3 (0.8)	1	..	..	1	..	..	0.001
	No	1284 (70.3)	450 (35.0)	190 (14.8)	220 (17.1)	411 (32.0)	13 (1.0)	0.82	0.62	1.07	0.55	0.40	0.76	
	Missing	170 (9.3)	36 (21.2)	44 (25.9)	36 (21.2)	44 (25.9)	10 (5.9)	..	..	..	..	..	..	
Worry	Yes	810 (44.4)	274 (33.8)	167 (20.6)	148 (18.3)	211 (26.0)	10 (1.2)	1	..	..	1	..	..	<0.001
	No	914 (50.1)	310 (33.9)	124 (13.6)	176 (19.3)	296 (32.4)	8 (0.9)	0.81	0.65	1.00	0.56	0.42	0.74	
	Missing	102 (5.6)	28 (27.5)	30 (29.4)	13 (12.7)	23 (22.5)	8 (7.8)	..	..	..	..	..	..	
Fear	Yes	303 (16.6)	90 (29.7)	77 (25.4)	62 (20.5)	72 (23.8)	2 (0.7)	1	..	..	1	..	..	0.023
	No	1404 (76.9)	482 (34.3)	228 (16.2)	257 (18.3)	422 (30.1)	15 (1.1)	0.99	0.74	1.34	0.65	0.47	0.90	
	Missing	119 (6.5)	40 (33.6)	16 (13.4)	18 (15.1)	36 (30.3)	9 (7.6)	..	..	..	..	..	..	
Loneliness	Yes	249 (13.6)	76 (30.5)	59 (23.7)	57 (22.9)	55 (22.1)	2 (0.8)	1	..	..	1	..	..	0.039
	No	1439 (78.8)	504 (35.0)	225 (15.6)	261 (18.1)	436 (30.3)	13 (0.9)	1.00	0.73	1.38	0.65	0.45	0.93	
	Missing	138 (7.6)	32 (23.2)	37 (26.8)	19 (13.8)	39 (28.3)	11 (8.0)	..	..	..	..	..	..	
Uncertainty	Yes	996 (54.5)	345 (34.6)	190 (19.1)	186 (18.7)	265 (26.6)	10 (1.0)	1	..	..	1	..	..	0.032
	No	778 (42.6)	247 (31.7)	121 (15.6)	145 (18.6)	256 (32.9)	9 (1.2)	0.78	0.63	0.97	0.75	0.57	0.99	
	Missing	52 (2.8)	20 (38.5)	10 (19.2)	6 (11.5)	9 (17.3)	7 (13.5)	..	..	..	..	..	..	

ORs are adjusted for age, sex and education level. Age is adjusted for sex and education level; sex is adjusted for age and education level; education level is adjusted for age and sex. * Analyses were performed considering mixed behaviors and unchanged as one category of reference. ** Young adults (18–44); middle-aged (45–64); aged (≥ 65).

**Table 4:** Odds ratio for type of change in alcohol drinking (decreased, increased, or unchanged) during COVID-19 lockdown by sociodemographic and work-related factors, health status, and lifestyle prior to lockdown, and psychological distress.

		**Total**	**Decreased**	**Increased**	**Unchanged**	**Missing**	**Decreased**			**Increased**			
		*n* (%)					ORs	95% CI		ORs	95% CI		
								lower limit	upper limit		lower limit	upper limit	*p* value
		1826 (100)	231 (12.6)	229 (12.5)	1275 (69.8)	91 (5.0)							
**Sociodemographic factors**													
Sex	Male	423 (23.2)	80 (18.9)	54 (12.8)	277 (65.5)	12 (2.8)	1	..	..	1	..	..	<0.001
	Female	1397(76.5)	151 (10.8)	174 (12.5)	997 (71.4)	75 (5.4)	0.47	0.34	0.65	0.80	0.57	1.13	
	Missing	6 (0.3)	0 (0.0)	1 (16.7)	1 (16.7)	4 (66.7)	..	..	..	..	..	..	
Age	Young adults (18–44)	818 (44.8)	162 (19.8)	131 (16.0)	503 (61.5)	22 (2.7)	1	..	..	1	..	..	<0.001
	Middle-aged (45–64)	802 (43.9)	56 (7.0)	94 (11.7)	607 (75.7)	45 (5.6)	0.27	0.20	0.39	0.60	0.44	0.80	
	Aged (≥ 65)	194 (10.6)	12 (6.2)	3 (1.5)	159 (82.0)	20 (10.3)	0.20	0.10	0.38	0.07	0.02	0.23	
	Missing	12 (0.7)	1 (8.3)	1 (8.3)	6 (50.0)	4 (33.3)	..	..	..	..	..	..	
Educational level	Low	94 (5.1)	6 (6.4)	9 (9.6)	68 (72.3)	11(11.7)	1	..	..	1	..	..	0.910
	Medium	805 (44.1)	97 (12.0)	95 (11.8)	573 (71.2)	40 (5.0)	1.46	0.60	3.57	0.91	0.43	1.93	
	High	889 (48.7)	124 (13.9)	123 (13.8)	610 (68.6)	32 (3.6)	1.41	0.58	3,44	0.97	0.46	2.05	
	Missing	38 (2.1)	4 (10.5)	2 (5.3)	24 (63.2)	8 (21.1)	..	..	..	..	..	..	
Household composition	Alone	208 (11.4)	36 (17.3)	19 (9.1)	140 (67.3)	13 (6.3)	1	..	..	1	..	..	0.012
	At least one cohabitant	1618 (88.6)	195 (12.1)	210 (13.0)	1135 (70.1)	78 (4.8)	0.55	0.36	0.85	1.17	0.70	1.96	
**Work-related factors**													
Changes in work modality	Work suspended	103 (5.6)	18 (17.5)	12 (11.7)	66 (64.1)	7 (6.8)	1	..	..	1	..	..	0.024
	More remote working	544 (29.8)	64 (11.8)	95 (17.5)	375 (68.9)	10 (1.8)	0.57	0.31	1.06	1.37	0.70	2.68	
	Unchanged	685 (37.5)	103 (15.0)	81 (11.8)	485 (70.8)	16 (2.3)	0.88	0.49	1.59	0.99	0.50	1.94	
	Not applicable	313 (17.1)	30 (9.6)	18 (5.8)	229 (73.2)	36 (11.5)	0.90	0.44	1.84	0.84	0.37	1.91	
	Missing	181 (9.9)	16 (8.8)	23 (12.7)	120 (66.3)	22 (12.2)	..	..	..	..	..	..	
**Health status and lifestyle prior to lockdown**													
Body mass index	Overweight	600 (32.9)	57 (9.5)	61 (10.2)	452 (75.3)	30 (5.0)	1	..	..	1	..	..	0.013
	Normal weight	1052 (57.6)	154 (14.6)	147 (14.0)	705 (67.0)	46 (4.4)	1.59	1.12	2.24	1.35	0.97	1.89	
	Missing	174 (9.5)	20 (11.5)	21 (12.1)	118 (67.8)	15 (8.6)	..	..	..	..	..	..	
Physical activity habits	Sedentary	239 (13.1)	27 (11.3)	26 (10.9)	176 (73.6)	10 (4.2)	1	..	..	1	..	..	0.037
	Partially active	995 (54.5)	110 (11.1)	120 (12.1)	729 (73.3)	36 (3.6)	0.97	0.61	1.55	1.15	0.72	1.82	
	Active	548 (30.0)	93 (17.0)	79 (14.4)	348 (63.5)	28 (5.1)	1.53	0.94	2.47	1.50	0.92	2.44	
	Missing	44 (2.4)	1 (2.3)	4 (9.1)	22 (50.0)	17 (38.6)	..	..	..	..	..	..	
Cigarette smoking habits	Smoker	389 (21.3)	59 (15.2)	60 (15.4)	253 (65.0)	17 (4.4)	1	..	..	1	..	..	0.123
	Non- smoker	1195 (65.4)	152 (12.7)	139 (11.6)	857 (71.7)	47 (3.9)	0.85	0.60	1.22	0.66	0.47	0.93	
	Former smoker	189 (10.4)	16 (8.5)	22 (11.6)	146 (77.2)	5 (2.6)	0.62	0.33	1.16	0.73	0.42	1.28	
	Missing	53 (2.9)	4 (7.5)	8 (15.1)	19 (35.8)	22 (41.5)	..	..	..	..	..	..	
**Psychological distress**													
Tension	Yes	359 (19.7)	43 (12.0)	74 (20.6)	232 (64.6)	10 (2.8)	1	..	..	1	..	..	<0.001
	No	1337 (73.2)	184 (13.8)	140 (10.5)	951 (71.1)	62 (4.6)	1.06	0.73	1.56	0.49	0.35	0.68	
	Missing	130 (7.1)	4 (3.1)	15 (11.5)	92 (70.8)	19 (14.6)	..	..	..	..	..	..	
Upset	Yes	372 (20.4)	45 (12.1)	72 (19.4)	246 (66.1)	9 (2.4)	1	..	..	1	..	..	0.001
	No	1284 (70.3)	181 (14.1)	143 (11.1)	900 (70.1)	60 (4.7)	1.00	0.69	1.44	0.54	0.39	0.75	
	Missing	170 (9.3)	5 (2.9)	14 (8.2)	129 (75.9)	22 (12.9)	..	..	..	..	..	..	
Worry	Yes	810 (44.4)	94 (11.6)	127 (15.7)	556 (68.6)	33 (4.1)	1	..	..	1	..	..	0.004
	No	914 (50.1)	133 (14.6)	95 (10.4)	647 (70.8)	39 (4.3)	1.04	0.77	1.42	0.61	0.45	0.82	
	Missing	102 (5.6)	4 (3.9)	7 (6.9)	72 (70.6)	19 (18.6)	..	..	..	..	..	..	
Fear	Yes	303 (16.6)	31 (10.2)	53 (17.5)	201 (66.3)	18 (5.9)	1	..	..	1	..	..	0.010
	No	1404 (76.9)	194 (13.8)	160 (11.4)	995 (70.9)	55 (3.9)	1.02	0.66	1.56	0.58	0.40	0.83	
	Missing	119 (6.5)	6 (5.0)	16 (13.4)	79 (66.4)	18 (15.1)	..	..	..	..	..	..	
Loneliness	Yes	249 (13.6)	47 (18.9)	41 (16.5)	152 (61.0)	9 (3.6)	1	..	..	1	..	..	0.001
	No	1439 (78.8)	180 (12.5)	170 (11.8)	1027 (71.4)	62 (4.3)	0.54	0.36	0.78	0.62	0.42	0.93	
	Missing	138 (7.6)	4 (2.9)	18 (13.0)	96 (69.6)	20 (14.5)	..	..	..	..	..	..	
Uncertainty	Yes	996 (54.5)	118 (11.8)	140 (14.1)	693 (69.6)	45 (4.5)	1	..	..	1	..	..	0.040
	No	778 (42.6)	107 (13.8)	78 (10.0)	558 (71.7)	35 (4.5)	1.06	0.79	1.43	0.69	0.51	0.93	
	Missing	52 (2.8)	6 (11.5)	11 (21.2)	24 (46.2)	11 (21.2)	..	..	..	..	..	..	

ORs are adjusted for age, sex and education level. Age is adjusted for sex and education level; sex is adjusted for age and education level; education level is adjusted for age and sex.

**Table 5:** Odds ratio for type of change in cigarette smoking (decreased, increased, or unchanged) during COVID-19 lockdown by sociodemographic and work-related factors, health status, and lifestyle prior to lockdown, and psychological distress.

		**Total**	**Decreased**	**Increased**	**Unchanged**	**Missing**	**Decreased**			**Increased**			
		*n* (%)					ORs	95% CI		ORs	95% CI		
								lower limit	upper limit		lower limit	upper limit	*p* value
		1826 (100)	75 (4.1)	140 (7.7)	1327 (72.7)	284 (15.6)							
**Sociodemographic factors**													
Sex	Male	423 (23.2)	18 (4.3)	32 (7.6)	311 (73.5)	62 (14.7)	1	..	..	1	..	..	0.827
	Female	1397(76.5)	57 (4.1)	108 (7.7)	1014 (72.6)	218 (15.6)	1.06	0.60	1.86	1.14	0.74	1.74	
	Missing	6 (0.3)	0 (0.0)	0 (0.0)	2 (33.3)	4 (66.7)	..	..	..	..	..	..	
Age	Young adult (18–44)	818 (44.8)	55 (6.7)	66 (8.1)	611 (74.7)	86 (10.5)	1	..	..	1	..	..	<0.001
	Middle-aged (45–64)	802 (43.9)	17 (2.1)	51 (6.4)	611 (76.2)	123 (15.3)	0.27	0.15	0.48	0.69	0.46	1.02	
	Aged (≥ 65)	194 (10.6)	2 (1.0)	23 (11.9)	99 (51.0)	70 (36.1)	0.22	0.05	0.91	2.11	1.23	3.62	
	Missing	12 (0.7)	1 (8.3)	0 (0.0)	6 (50.0)	5 (41.7)	..	..	..	..	..	..	
Educational level	Low	94 (5.1)	2 (2.1)	7 (7.4)	59 (62.8)	26 (27.7)	1	..	..	1	..	..	0.004
	Medium	805 (44.1)	40 (5.0)	73 (9.1)	545 (67.7)	147 (18.3)	1.58	0.37	6.87	1.34	0.57	3.11	
	High	889 (48.7)	32 (3.6)	58 (6.5)	700 (78.7)	99 (11.1)	0.76	0.17	3.37	0.77	0.33	1.83	
	Missing	38 (2.1)	1 (2.6)	2 (5.3)	23 (60.5)	12 (31.6)	..	..	..	..	..	..	
Household composition	Alone	208 (11.4)	12 (5.8)	21 (10.1)	138 (66.3)	37 (17.8)	1	..	..	1	..	..	0.027
	At least one cohabitant	1618 (88.6)	63 (3.9)	119 (7.4)	1189 (73.5)	247 (15.3)	0.48	0.25	0.93	0.63	0.38	1.05	
**Work-related factors**													
Changes in work modality	Work suspended	103 (5.6)	6 (5.8)	10 (9.7)	72 (69.9)	15 (14.6)	1	..	..	1	..	..	0.126
	More remote working	544 (29.8)	26 (4.8)	41 (7.5)	419 (77.0)	58 (10.7)	0.80	0.31	2.09	0.80	0.38	1.69	
	Unchanged	685 (37.5)	27 (3.9)	62 (9.1)	515 (75.2)	81 (11.8)	0.84	0.33	2.15	0.89	0.43	1.84	
	Not applicable	313 (17.1)	9 (2.9)	17 (5.4)	197 (62.9)	90 (28.8)	0.97	0.32	3.00	0.30	0.11	0.78	
	Missing	181 (9.9)	7 (3.9)	10 (5.5)	124 (68.5)	40 (22.1)	..	..	..	..	..	..	
**Health status and lifestyle prior to lockdown**													
Body mass index	Overweight	600 (32.9)	22 (3.7)	39 (6.5)	434 (72.3)	105 (17.5)	1	..	..	1	..	..	0.658
	Normal weight	1052 (57.6)	45 (4.3)	81 (7.7)	785 (74.6)	141 (13.4)	1.03	0.60	1.79	1.21	0.80	1.84	
	Missing	174 (9.5)	8 (4.6)	20 (11.5)	108 (62.1)	38 (21.8)	..	..	..	..	..	..	
Physical activity habits	Sedentary	239 (13.1)	12 (5.0)	14 (5.9)	176 (73.6)	37 (15.5)	1	..	..	1	..	..	0.281
	Partially active	995 (54.5)	31 (3.1)	79 (7.9)	733 (73.7)	152 (15.3)	0.60	0.30	1.21	1.30	0.72	2.37	
	Active	548 (30.0)	30 (5.5)	45 (8.2)	398 (72.6)	75 (13.7)	0.99	0.49	2.02	1.39	0.74	2.62	
	Missing	44 (2.4)	2 (4.5)	2 (4.5)	20 (45.5)	20 (45.5)	..	..	..	..	..	..	
Cigarette smoking habits	Smoker	389 (21.3)	72 (18.5)	127 (32.6)	171 (44.0)	19 (4.9)	1	..	..	1	..	..	<0.001
	Non-smoker	1195 (65.4)	2 (0.2)	6 (0.5)	987 (82.6)	200 (16.7)	0.00	0.00	0.02	0.01	0.00	0.02	
	Former smoker	189 (10.4)	0 (0.0)	5 (2.6)	156 (82.5)	28 (14.8)	-	0	-	0.04	0.02	0.11	
	Missing	53 (2.9)	1 (1.9)	2 (3.8)	13 (24.5)	37 (69.8)	..	..	..	..	..	..	
**Psychological distress**													
Tension	Yes	359 (19.7)	17 (4.7)	37 (10.3)	248 (69.1)	57 (15.9)	1	..	..	1	..	..	0.129
	No	1337 (73.2)	55 (4.1)	97 (7.3)	1006 (75.2)	179 (13.4)	0.90	0.50	1.60	0.65	0.43	0.99	
	Missing	130 (7.1)	3 (2.3)	6 (4.6)	73 (56.2)	48 (36.9)	..	..	..	..	..	..	
Upset	Yes	372 (20.4)	15 (4.0)	39 (10.5)	264 (71.0)	54 (14.5)	1	..	..	1	..	..	0.034
	No	1284 (70.3)	55 (4.3)	84 (6.5)	975 (75.9)	170 (13.2)	1.02	0.56	1.87	0.58	0.38	0.88	
	Missing	170 (9.3)	5 (2.9)	17 (10.0)	88 (51.8)	60 (35.3)	..	..	..	..	..	..	
Worry	Yes	810 (44.4)	31 (3.8)	64 (7.9)	595 (73.5)	120 (14.8)	1	..	..	1	..	..	0.918
	No	914 (50.1)	40 (4.4)	69 (7.5)	677 (74.1)	128 (14.0)	1.11	0.67	1.84	1.00	0.69	1.45	
	Missing	102 (5.6)	4 (3.9)	7 (6.9)	55 (53.9)	36 (35.3)	..	..	..	..	..	..	
Fear	Yes	303 (16.6)	9 (3.0)	30 (9.9)	209 (69.0)	55 (18.2)	1	..	..	1	..	..	0.224
	No	1404 (76.9)	62 (4.4)	105 (7.5)	1047 (74.6)	190 (13.5)	1.36	0.65	2.83	0.72	0.46	1.12	
	Missing	119 (6.5)	4 (3.4)	5 (4.2)	71 (59.7)	39 (32.8)	..	..	..	..	..	..	
Loneliness	Yes	249 (13.6)	17 (6.8)	28 (11.2)	166 (66.7)	38 (15.3)	1	..	..	1	..	..	0.027
	No	1439 (78.8)	53(3.7)	105 (7.3)	1089 (75.7)	192 (13.3)	0.55	0.31	1.01	0.61	0.39	0.97	
	Missing	138 (7.6)	5 (3.6)	7 (5.1)	72 (52.2)	54 (39.1)	..	..	..	..	..	..	
Uncertainty	Yes	996 (54.5)	40 (4.0)	98 (9.8)	725 (72.8)	133 (13.4)	1	..	..	1	..	..	0.011
	No	778 (42.6)	32 (4.1)	41 (5.3)	574 (73.8)	131 (16.8)	1.05	0.64	1.72	0.56	0.38	0.82	
	Missing	52 (2.8)	3 (5.8)	1 (1.9)	28 (53.8)	20 (38.5)	..	..	..	..	..	..	

ORs are adjusted for age, sex and education level. Age is adjusted for sex and education level; sex is adjusted for age and education level; education level is adjusted for age and sex.

## Data Availability

The dataset generated and analysed is retained by the Information and Technologies Service of the Azienda USL-IRCCS of Reggio Emilia and is available upon request from the corresponding author.

## Supplementary Materials

The following are available online at https://www.mdpi.com/article/10.3390/nu13051600/s1, Supplementary Material S1. Questionnaire on lifestyle changes in the general population of the province of Reggio Emilia following the COVID-19 lockdown. Supplementary Material S2. The co-occurrence of more than one negative change in lifestyle. Supplementary Material S3. Sociodemographic and work-related factors, health status, and lifestyle prior to lockdown, and psychological distress associated with changes in lifestyle.
