# Supplementary material for: Beyond Lockdown: The Potential Side Effects of the SARS-CoV-2 Pandemic on Public Health
Source: Nutrients. 2021 May 11;13(5):1600. doi: 10.3390/nu13051600 (PMC8170902; doi:10.3390/nu13051600)
Supplement: Supplementary file 1 [file nutrients-13-01600-s001.zip › nutrients-1166274-supplementary.pdf]

S1. QUESTIONNAIRE ON LIFESTYLE CHANGES IN THE GENERAL POPULATION OF THE PROVINCE OF REGGIO EMILIA FOLLOWING THE COVID-19 LOCKDOWN

SOCIODEMOGRAPHIC FACTORS

1. **Sex**

F	M

2. **Age**

18-23	24-29	30-35	36-41	42-47	48-53	54-59	60-65	66-71	>71

3. **CITY OF RESIDENCE:** ___________________

4. **Postal code:** _________________

5. **Citizenship:**
- Italian
- Foreign

6. **Marital status:**
- Married
- Cohabitation
- Unmarried
- Widowed
- Divorced
- Separated

7. **Education level**
- None
- Primary school
- Middle school
- High school
- University degree
- Post-university degree

8. **Current home size**
- < 50 m^2
- 50 - 100 m^2
- > 100 m^2

9. **Does your home have any outdoor areas?**
- Garden
- Terrace
- Balcony
- Other
- None

10. **Do you have any children?**

- No
- Yes, at least one child under the age of 12
- Yes, all children over the age of 12

11. **Who do you live with? (multiple choice)**

- Alone
- With partner
- With child/children
- With parents
- With a family member with a serious disability (child/parent)
- With several family members with serious disabilities (children or parents)
- With other relatives or cohabitants

12. **In total, how many people live with you (excluding yourself)? :________**

13. **Are you in isolation after testing positive for COVID-19?**

- Yes
- No

14. **If so, what kind of isolation?**

- Own home
- Hotel
- Rems (COVID-19 residential facility)
- Other temporary home

WORK-RELATED FACTORS

15. **Before the COVID-19 emergency, what was your employment status?**

- Employee in the public sector
- Employee in the private sector
- Self-employed
- Student
- Retired
- Unemployed
- Housewife
- Other (specify): ___________________

16. **Before the COVID-19 emergency, where did you work?**

- At my workplace
- From home
- Both from home and at my workplace

17. **Current employment status**

- Continue to work
- On vacation leave
- Leave of absence
- Unemployment insurance
- Business closed due to the COVID-19 emergency
- Other

18. **If you work or study, how do you do these activities?**

- My job has been suspended
- I go to work
- From home

- I work both from home and at my workplace
- I follow class lectures from home

19. **Are you currently experiencing economic problems?**
    - No difficulties
    - Some difficulties
    - Many difficulties

USE OF DIGITAL DEVICES DATA

20. **Do you currently have a suitable Internet connection?**

- Yes
- No
- I don't know

21. **Do you currently have any IT devices, such as a computer, tablet, and/ or smartphone, and a printer?**

- Yes
- No
- I don't know

22. **How do you evaluate your computer literacy?**

- Poor
- Sufficient
- Good
- Excellent

LIFESTYLE CHANGES, ANTRHOPOMETRIC DATA AND HEALTH STATUS

23. **Weight:____**
24. **Height:______**
25. **Do you have any disease-related fee exemptions?**

- Yes
- No

26. **If exempt, for which disease? (optional question)**

27. **Before the COVID-19 emergency, how did you consider yourself?**

- Active (a person who has a physically demanding job that requires considerable physical effort and/or 30 minutes of moderate physical activity at least 5 days a week and/or intense activity for more than 20 minutes for at least 3 days a week)
- Partially active (a person who does not do a physically demanding job but who does some physical activity in his/ her free time)
- Sedentary (a person who neither has a physically demanding job nor does any physical activity in his/ her free time)

28. **If you were active or partially active, how many days per week?**

- 1-2 days / week
- 3-4 days / week
- > 4 days / week

29. **Currently, how do you consider yourself?**

- Active (a person who has a physically demanding job that requires considerable physical effort and/or 30 minutes of moderate physical activity at least 5 days a week and/or intense activity for more than 20 minutes for at least 3 days a week)
- Partially active (a person who does not do a physically demanding job but who does some physical activity in his/ her free time)
- Sedentary (a person who neither has a physically demanding job nor does any physical activity in his/ her free time)

30. **If you are active or partially active, how many days per week?**

- 1-2 days / week
- 3-4 days / week
- > 4 days / week

31. **What kind of physical activity do you currently do?**

- Treadmill / running / walking / stairs
- Stationary bike / bicycling
- Pilates / Yoga / stretching / free body / gym
- Gardening
- Other:____________________

32. **What kind of IT do you use to perform physical activity at home?**

- Group or one-to-one video chat
- Online courses/ guides
- Applications on smartphones, tablets, computer
- Programs from your trainer
- Alone
- Other

33. **Has your diet changed (improve/ worsen in terms of the consumption of some foods, meal times, weight control)?**

- Yes
- No
- I don’t know

34. **If so, what changes have occurred? (multiple choice)**

- I have add snacks, sweets, carbonated drinks
- I eat more abundant main meals (breakfast and/or lunch and/or dinner)
- I eat at more regular times
- I pay more attention to eating healthier (i.e. quality and/or variety and/or meal cooking)
- I eat more prepackaged foods
- Before COVID-19, I was forced to often eat out

35. **Before the COVID-19 emergency, how many portions of fruit or vegetables did you eat daily?**

(portion means a quantity of raw fruit or vegetables that can be held in the palm of your hand, or half a plate of cooked vegetables)

- No portion of fruit or vegetables
- 1-2 portions / day
- 3-4 portions / day
- 5-6 portions / day
- > 6 portions / day

36. **Following the COVID-19 emergency, how has your consumption of fruit and vegetables changed?**

- Increased

- Decreased
- Unchanged
- I don’t eat fruit or vegetables

37. **Do you currently consider your diet healthy?**

- Yes
- No
- I don’t know

38. Before the COVID-19 emergency, how many alcoholic drinks did you consume? (The alcoholic unit (UA) corresponds to 12 grams of ethanol, an amount approximately contained in a can of beer (330 ml at 4.5 °), a glass of wine (125 ml at 12 °), a glass of liqueur (40 ml at 40 °), or an aperitif (80 ml at 38 °).

- None
- < 2 alcoholic unit (UA) on average per day
- ≥2 alcoholic unit (UA) on average per day

39. **Following the COVID-19 emergency, how has your consumption of alcohol changed?**

- Increased
- Decreased
- Unchanged
- I don't drink alcohol

40. **Before the emergency COVID-19, how did you consider yourself in terms of smoking?**

- Non-smoker (a person who claims to have smoked less than 100 cigarettes in his/ her life (5 packs of 20) and is not currently a smoker)
- Smoker (a person who claims to have smoked at least 100 cigarettes in his/ her life (5 packs of 20) and to be a smoker at the time of the interview or to have stopped smoking less than 6 months ago)
- Occasional smoker (a person who declares to not smoke every day)
- Daily smoker (a person who claims to smoke at least one cigarette every day).
- Ex-smoker (a person who claims to have smoked at least 100 cigarettes in his/ her life (5 packs of 20), to NOT be a smoker at the time of the interview, and to have stopped smoking at least 6 months ago)
- Abstention smoker (a person who claims to have quit smoking less than 6 months ago)

41. **Following the COVID-19 emergency, how has your consumption of cigarettes changed?**

- Increased
- Decreased
- Unchanged
- I’m not a smoker

42. **Have your sleep habits changed during lockdown (sleep quantity and regularity)?**

- Yes
- No
- I don’t know

43. **How do you consider your sleep quality now?**

- Very good
- Good
- Not very good
- Not good at all

44. **What are the new activities that you are doing that you did not do before COVID-19?**

- Reading
- Resting
- Cooking
- Watching TV
- Using social media
- Dedicating time to family activities
- Gardening / housecleaning
- Volunteer work
- None
- Other: ___________________

45. **In the future, are you going to maintain your acquired lifestyle habits or return to your previous behaviors?**

**a) Physical activity**	maintain	return	I don't know/ unchanged
**b) Eating habits**	maintain	return	I don't know/ unchanged
**c) Smoking, drinking habits**	maintain	return	I don't know/ unchanged
**d) Sleep habits**	maintain	return	I don't know/ unchanged
**e) Leisure activities**	maintain	return	I don't know/ unchanged

USE OF SUPPORT SERVICES OF THE TERRITORIAL NETWORK DATA

46. **Have you used/ Are you using any of the support made available by the municipality / associations / local health authorities? (multiple choice)**

- Home delivery of groceries
- Home delivery of medications
- Information hotline
- Mental health hotline
- Economic aid, e.g. food vouchers
- Area facilities for the care of animals
- Activation of social services
- Other: ___________________

47. What kind of support would you have liked in order to deal **with this situation but which was not available?** (Open question)

____

____

48. **What/ who helped you to overcome the challenges of lockdown? (multiple choice)**

- family or friends
- volunteers / neighbors
- healthcare professionals
- the municipality or local associations
- leisure activities (i.e. reading, garden care, etc.)
- continuing to work
- optimistic attitude
- physical activity
- drinking and / or smoking

- taking care of pets
- salary
- drugs
- other

EMOTIONAL STATE

49. At this moment, are you feeling?

**Nervous**	1. Not at all	2. A little	3. Quite	4. A lot
**Upset**	1. Not at all	2. A little	3. Quite	4. A lot
**Worried**	1. Not at all	2. A little	3. Quite	4. A lot
**Afraid**	1. Not at all	2. A little	3. Quite	4. A lot
**Lonely**	1. Not at all	2. A little	3. Quite	4. A lot
**Uncertain about the future**	1. Not at all	2. A little	3. Quite	4. A lot

S2. The co-occurrence of more than one negative change in lifestyle

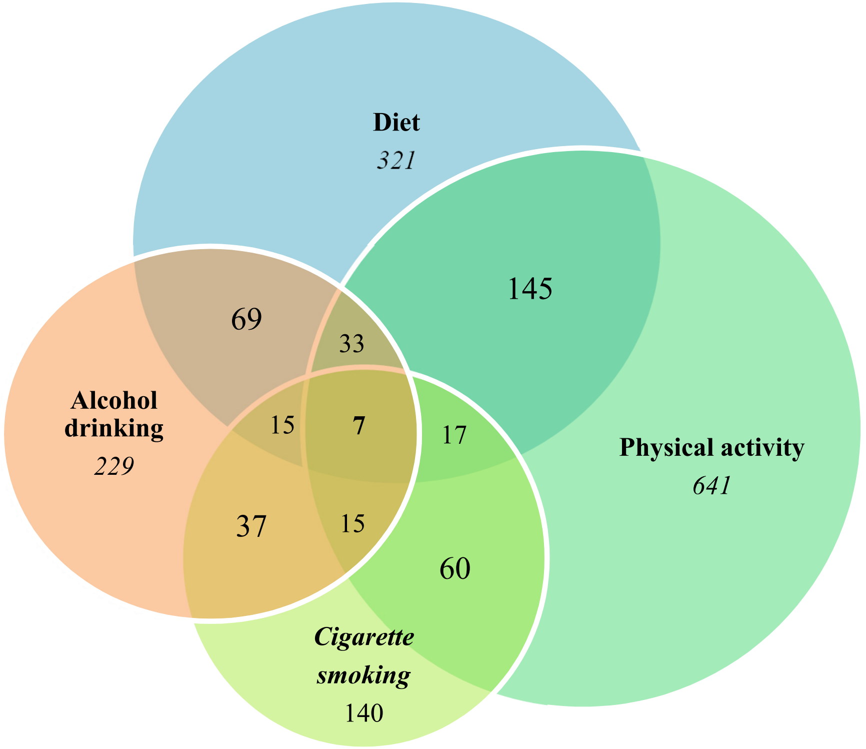

***S3*. Sociodemographic and work-related factors, health status, and lifestyle prior to lockdown, and psychological distress associated with changes in lifestyle**

<table>
<tr><th colspan="2"></th><th>Physical activity</th><th>Diet</th><th>Alcohol drinking</th><th>Cigarette smoking</th></tr>
<tr><th colspan="2">Potential exposures</th><td colspan="4"></td></tr>
<tr><th rowspan="5">Sociodemographic factors</th><td rowspan="1">Sex</td><td>Females were more likely to improve compared to males</td><td>Females were more likely to worsen compared to males</td><td>Females were less likely to decrease compared to males</td><td>The determinant did not affect this lifestyle</td></tr>
<tr><td rowspan="2">Age*</td><td>Middle aged and aged were less likely to improve compared to young adults</td><td>Middle aged were less likely to worsen compared to young adults</td><td rowspan="2">Middle Aged and aged were less likely both to increase and decrease compared to young adults</td><td rowspan="2">Middle aged were less likely to decrease and aged were more likely to increase compared to young adults</td></tr>
<tr><td>Aged were less likely to worsen compared to young adults</td><td>Aged were less likely to improve compared to young adults</td></tr>
<tr><td>Education level</td><td>The determinant did not affect this lifestyle</td><td>Participants with medium and high education levels were more likely to improve compared to those with low education level</td><td>The determinant did not affect this lifestyle</td><td>Participants with medium education level were more likely both to increase and decrease compared to those with low education level</td></tr>
<tr><td>Household composition</td><td>The determinant did not affect this lifestyle</td><td>The determinant did not affect this lifestyle</td><td>Participants who lived with at least one cohabitant were less likely to decrease compared to those who lived alone</td><td>Participants who lived with at least one cohabitant were less likely to decrease compared to those who lived alone</td></tr>
<tr><th rowspan="2">Work-related factors</th><td rowspan="2">Changes in work modality</td><td>Remote working led to a lower likelihood of worsening than who suspended work activity</td><td>Remote working led to a lower likelihood of worsening diet compared to those whose work was suspended</td><td rowspan="2">The determinant did not affect this lifestyle</td><td rowspan="2">The determinant did not affect this lifestyle</td></tr>
<tr><td>Unchanged work activity led to a lower likelihood of worsening than who suspended work activity</td><td>Unchanged work activity led to a lower likelihood of worsening compared to those whose work was suspended</td></tr>
</table>

**Health status and lifestyle prior to lockdown**	*Body mass index*	**Normal weight** participants were less likely to improve compared to those who were overweight	**Normal weight** participants were less likely to worsen compared to those who were overweight	**Normal weight** participants were more likely to decrease compared to those who were overweight	The determinant did not affect this lifestyle
	Physical activity habits	Participants who were **partially active** were less likely to improve compared to those who were sedentary	The determinant did not affect this lifestyle	Participants who were **active** were more likely both to increase and decrease compared to those who were sedentary	The determinant did not affect this lifestyle
	Cigarette smoking habits	Participants who were **non-smokers** were less likely to improve compared to smokers	The determinant did not affect this lifestyle	The determinant did not affect this lifestyle	Participants who were **non-smokers and former smokers** were less likely both to increase and decrease compared to smokers
**Symptoms of Psychological distress ****	*Tension*<br>*Upset*<br>*Worry*<br>*Fear*<br>*Loneliness*<br>*Uncertainty*	Participants who reported **no psychological distress** were less likely to worsen compared to those who reported psychological vulnerability	Participants who reported **no psychological distress** were less likely to worsen compared to those who reported psychological vulnerability	Participants who reported **no psychological distress** were less likely to increase compared to those who reported psychological vulnerability	Participants who reported **no psychological distress** were less likely to increase compared to those who reported psychological vulnerability

*Young adults (18-44); Middle-aged (45-64); Aged (≥ 65)

**With respect to psychological distress, Fear did not affect changes in physical activity, and both Fear and Worry did not affect smoking-related behaviours. Moreover, participants who did not report Uncertainty were also less likely to improve diet. Finally, people who did not report Loneliness were also less likely to decrease alcohol drinking.
